# Poor adherence to neonatal resuscitation guidelines exposed; an observational study using camera surveillance at a tertiary hospital in Nepal

**DOI:** 10.1186/1471-2431-14-233

**Published:** 2014-09-16

**Authors:** Caroline Lindbäck, Ashish KC, Johan Wrammert, Ravi Vitrakoti, Uwe Ewald, Mats Målqvist

**Affiliations:** International Maternal and Child Health, Department of Women’s and Children’s Health, Uppsala University, Uppsala, Sweden; UNICEF, Kathmandu, Nepal; Paropakar Women’s and Maternity Hospital, Kathmandu, Nepal

**Keywords:** Infant, Newborn, Asphyxia neonatorum, Neonatal mortality, Resuscitation, Noninvasive ventilation, Developing countries, Nepal, Video recording, Reliability of results

## Abstract

**Background:**

Each year an estimated 10 million newborns require assistance to initiate breathing, and about 900 000 die due to intrapartum-related complications. Further research is required in several areas concerning neonatal resuscitation, particularly in settings with limited resources where the highest proportion of intrapartum-related deaths occur. The aim of this study is to use CCD-camera recordings to evaluate resuscitation routines at a tertiary hospital in Nepal.

**Methods:**

CCD-cameras recorded the resuscitations taking place and CCD-observational record forms were completed for each case. The resuscitation routines were then assessed and compared with existing guidelines. To evaluate the reliability of the observational form, 50 films were randomly selected and two independent observers completed two sets of forms for each case. The results were then cross-compared.

**Results:**

During the study period 1827 newborns were taken to the resuscitation table, and more than half of them (53.3%) were noted as not crying prior to resuscitation.

Suction was used in almost 90% of newborns brought to the resuscitation table, whereas bag-and-mask ventilation was only used in less than 10%. The chance to receive ventilation with bag-and-mask for a newborn not crying when brought to the resuscitation table was higher for boys (AdjOR 1.44), low birth weight babies (AdjOR 1.68) and babies that were delivered by caesarean section (AdjOR 1.64).

The reliability of the observational form varied considerably amongst the different variables analyzed, but was high for all variables concerning the use of bag-and-mask ventilation and the variable whether suction was used or not, all matching in over 91% of the forms.

**Conclusions:**

CCD camera technique was a feasible method to assess resuscitation practices in this low resource hospital setting. In most aspects, the staff did not adhere to guidelines regarding neonatal resuscitation. The use of bag-and-mask ventilation was inadequate, and suction was given excessively in terms of protocol. Further studies exploring the underlying causes behind the lack of adherence to the neonatal resuscitation guidelines should be conducted.

**Electronic supplementary material:**

The online version of this article (doi:10.1186/1471-2431-14-233) contains supplementary material, which is available to authorized users.

## Background

Each year, an estimated 10 million newborns require assistance to initiate breathing, and about 900 000 of these die due to intrapartum-related complications, previously termed “birth asphyxia” [1]. While intrapartum-related neonatal deaths account for 9% of all under-five mortality, a proportion larger than malaria (7%), this issue has received relatively low attention [2]. About 5-10% of babies do not spontaneously breathe at birth and require some degree of assistance. In most cases basic resuscitation such as stimulation, airway cleaning, drying, warmth, and in some cases bag-and-mask ventilation will be sufficient. Only about 2% of all babies who do not breathe at birth require more advanced resuscitation, such as medications, intubation, or chest compressions [1].

Low-income countries have the highest proportion of intrapartum-related deaths, and over a third are found in South East Asia alone [1]. A majority of these babies could be saved using relatively inexpensive interventions, such as low-technology community-based interventions, and providing skilled care at birth [3]. In South East Asia, only 34% (in year 2000–2007) of health personnel in birth facilities are trained as skilled birth attendants. Studies have shown that providing basic neonatal resuscitation training at birth facilities in low and middle income countries reduce deaths related to birth asphyxiation by an average of about 30% [1], and the need for clinical guidelines on basic newborn resuscitation suitable for settings with limited resources is universally recognized [4]. A basic protocol regarding neonatal resuscitation in low-resource settings can be obtained in the World Health Organization (WHO)’s Pocket Book of Hospital Care for Children, issued in 2005. In general, this states that if a baby is not breathing properly after 30 seconds of initial drying, stimulation, and clearing of the airways (when necessary), bag-and-mask ventilation should be initiated [5]. In 2012, the WHO published updated international recommendations for neonatal resuscitation. While some elements contained in these guidelines have a strong strength of recommendation, in almost half of them (9/19) the strength of recommendation is considered low. For a majority of the recommendations (15/19), the evidence supporting them was considered to be of low or very low quality, or lacking entirely. Thus, further research is required in several areas concerning neonatal resuscitation in settings with limited resources [4].

Camera recordings as a mean to evaluate and improve performance in emergency medicine was first reported in 1969 by Peliter et al. In 1988, an article by Hoyt et al. demonstrated successful use of video recordings when aiming to improve the staff performance in trauma resuscitation [6]. However, the majority of published work dealing with resuscitation issues is based on medical records, and most are only regarding the adult population [7]. Before the year 2000 there had been no reports published concerning the use of similar camera recordings when assessing neonatal resuscitation [6]. Since then, only a few articles have been published on the matter, although these have mostly generated positive results when using camera recordings as a way of assessing neonatal resuscitation [6, 8–10]. It can provide important information used for quality assessment and education as well as improving teamwork, leadership and communication within a resuscitation workgroup. When analyzing resuscitation recordings it has been reported that in over 50% of these some deviation from current guidelines could be identified. By recognizing reoccurring errors, preventative measures can be implemented in an effort to correct them [10]. Video recordings have great potential in terms of optimizing newborn resuscitation, and therefore further studies are needed to assess the validity and reliability when used in clinical practice [6, 11].

The aim of the present study is to use Dome change-coupled device (CCD) Camera recordings to evaluate resuscitation routines at a tertiary hospital in Nepal.

## Methods

### Setting

This cross-sectional study is a sub-study within a collaborative project between Uppsala University, Paropakar Maternity and Women’s Hospital (PMWH) in Kathmandu, and the Ministry of Health and Population in Nepal. The aim of the main project is to evaluate and improve neonatal resuscitation and survival in a tertiary hospital in Kathmandu [12]. This will be achieved by using the Helping Babies Breathe (HBB) protocol; a neonatal resuscitation guideline developed in association with the American Academy of Paediatrics, designed to train birth attendants in developing countries the essential skill of new born resuscitation [13]. PMWH is a tertiary government hospital and works as a central referral hospital of the country, providing gynaecological and obstetric services. The hospital has just over 23 000 deliveries per year, which gives an average delivery rate at about 63 babies per day. The perinatal mortality rate (PMR) at the hospital is currently about 30 per 1,000 live births, with an early neonatal mortality rate (death in the first 7 days of life) of 9 per 1,000 and a stillbirth rate of 19 per 1,000 pregnancies. Abnormal deliveries account for 26% - 28% of the total deliveries, and the caesarean rate is about 17% [14].

### Data collection

Budget Dome change-coupled device (CCD) Cameras (model no. MTC-505DH) were used to collect data on the hospital’s neonatal resuscitation routines. A camera was placed at each of the 6 resuscitation tables in the hospital, arranged accordingly: one in the Operating Theatre, one in the maternal and newborn service centre (MNSC), one in the emergency admission room and three in the labour rooms. The cameras had a progressive scan sensor and excellent low light performance. The cameras were equipped with motion sensors that recorded all movement within the camera’s field of vision. All film material recorded were sent to and stored on the main computer for data collection. Material captured by the CCD- cameras that did not contain a resuscitation situation, such as equipment checks, babies placed on the table for other reasons than resuscitation, staff using the table as support for updating medical records etc., where reviewed and disregarded as disturbance.

Two separate forms were uniquely developed for this study: a Case Record Form and a CCD Observation Record Form. Time and place of the resuscitations recorded by the CCD-cameras were captured. This was then matched to medical records and the Case Record Form, which contained the mother’s name, identification- and admission number. This information was transferred to the CCD Observation Record Form (Additional file 1), where a total of 12 sections had to be completed using information obtained from the Case Record Forms. In addition to the mother’s name, ID- and admission number, these included date, time, and place of birth, the baby’s gestational age, Apgar-score at one and five minutes, birth weight, sex, and whether the baby was referred or not after the resuscitation.

The CCD Observation Record Form was then used to register information from the corresponding resuscitation case recorded by the CCD-cameras. Surveillance officers, not in other ways connected to the hospital or the staff, were trained in how to use the data collection software and how to fill in the Observation Forms. The form had 14 sections to be filled out regarding observations made when watching the camera recordings. These sections included place, date, and time of resuscitation, whether the baby was crying when resuscitation was initiated, which specific resuscitation techniques were used (i.e. stimulation, suction, oxygen and ventilation) and the time intervals of which they were performed, time of first cry, and outcome. The surveillance officers then had to sign the Observation Forms and hand them to the staff in charge of data management.

### Data management

A data entry officer transferred the completed CCD Observation Forms into The Census and Survey Processing System (CSPro), a public domain software package developed and supported by the U.S. Census Bureau and ICF Macro, which is interfaced with Statistical Package for the Social Sciences (SPSS 12.0).

### Data analysis

The data collected from resuscitation cases recorded between July 1^st^ and October 31^st^ 2012 were analyzed to assess the resuscitation routines at the hospital. These were then compared with existing guidelines. Relations between the use of bag and mask ventilation and factors such as sex, birth weight, mode, and time of delivery and were also analyzed. In order for future comparison before and after HBB-intervention, Apgar-scores were extracted from medical records and analyzed in terms of resuscitation techniques used.

50 CCD-camera recordings were randomly selected out of 257 recorded in October and November 2012. To evaluate the inter- and intra-rater reliability of the observational forms, two independent observers completed two sets of forms for each case, with roughly 6 weeks in-between the two viewings conducted by each observer. Out of the 14 original sections in the Observational form, 12 were used when analyzing the reliability. Place and date of the resuscitation were filled out in order to match the two sets of forms together, and thus were not included in the analysis. Using the time intervals given for each of the four individual resuscitation techniques, the total time each technique was performed was calculated and analyzed as four additional variables. The total time from when the baby was placed at the resuscitation table until the first cry was also calculated. In total there were 17 variables individually analyzed and cross-compared in each resuscitation case. Analyses were made in SPSS 12.0. A confidence level of 95% was considered significant.

### Ethical approval

CCD cameras were mounted at all resuscitation tables with the permission of hospital management. All babies brought to the tables were recorded. Because of logistic concerns, since it being impossible to predict who needed resuscitation, written informed consent was obtained from all parents of referent population before discharge [12]. The video material for referent babies was thereafter edited and stored in a safe location and was only utilized by the researchers. The study was approved by the Nepal Health Research Council (reg. No 37/2012) and the Ethical Review Board of Uppsala University (dnr 2012/267).

## Results

**Figure 1:**
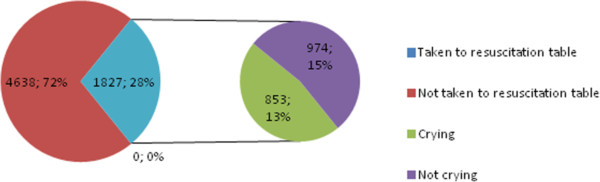
**Newborns taken to the resuscitation table.** The number and % of newborns that were taken to the resuscitation table, crying and not crying, out of the total number of babies born during the delivery period (6465).

Between July 1^st^ and October 31^st^ 2012 there were 6465 deliveries taking place at the study site. Out of these, 28 percent of the newborns (1827) were taken to the resuscitation table and captured by surveillance cameras. More than half of the newborns (53.3%) brought to the resuscitation table were noted as not crying prior to resuscitation (Figure 1).

The most frequent action taken was suction of the airways, both for crying (85.0%) as well as for non-crying babies (92.3%) (Table 1). Ventilation with bag-and-mask was only performed on 172 (9.4%) of the recorded cases, whereof 162 were noted as not crying when brought to the resuscitation table.

**Table 1:** **Actions taken for newborns brought to the resuscitation table**

	All newborns brought to table		Newborns crying when brought to table		Newborns not crying when brought to table	
	n	***%***	n	***%***	n	***%***
**Stimulation**						
Yes	824	*45.1*	197	*23.1*	627	*64.4*
No	1003	*54.9*	656	*76.9*	347	*35.6*
**Bag and mask**						
Yes	172	*9.4*	10	*1.2*	162	*16.6*
No	1655	*90.6*	843	*98.8*	812	*83.4*
**Suction performed**						
Yes	1624	*88.9*	725	*85.0*	899	*92.3*
No	203	*11.1*	128	*15.0*	75	*7.7*
**Oxygen provided**						
Yes	687	*37.6*	175	*20.5*	512	*52.6*
No	1140	*62.4*	678	*79.5*	462	*47.4*

The mean birth weight for recorded cases was 2860 grams, with non-crying newborns being lighter (2910 gram vs 2817 grams, p < 0.01). The low birth weight rate in the sample was 16.9% (308/1827) and 56% (1031/1827) of recorded cases were boys. The chance to receive ventilation with bag-and-mask for a newborn not crying when brought to the resuscitation table was higher for boys and low birth weight babies (AdjOR 1.44 and 1.68 respectively). There was no difference in ventilation if the baby was born at night-time (8 pm-8 am), but there was an increased chance for a non-breathing baby to be ventilated if the delivery was done by caesarean section (AdjOR 1.64) (Table 2).

**Table 2:** **Multivariate logistic regression model displaying the chance of receiving ventilation for newborns not crying when brought to resuscitation table (n = 974)**

	Receiving ventilation with B&M	Not receiving ventilation with B&M	Adj odds ratio (CI 95%)	p-value
**Sex**	n	n		
Girl	54	348	Ref	
Boy	108	464	1.44 (1.01-2.06)	0.05
**Birth weight**				
Normal birth weight (≥2500 grams)	117	663	Ref	
Low birth weight (<2500 grams)	45	149	1.68 (1.13-2.51)	0.01
**Mode of delivery**				
Vaginal	105	616	Ref	
Caesarean section	57	196	1.64 (1.14-2.36)	0.01
**Time of delivery**				
Day (8 am-8 pm)	100	465	Ref	
Night (8 pm – 8 am)	62	347	0.83 (0.59-1.18)	0.30

**Figure 2:**
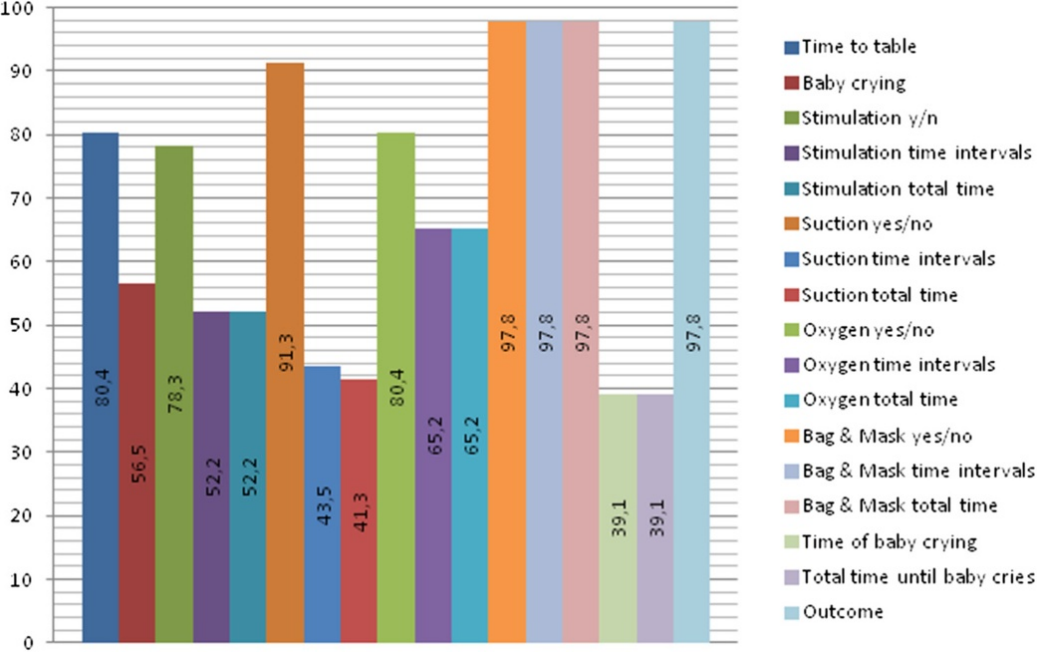
**All forms – variables matching (%).** The % of the specific variables matching across all entries for the same resuscitation case, all forms considered.

Apgar-scores were recorded one and five minutes after birth. Median Apgar-score after one minute was 5/10 and after five minutes 7/10. There were 198 babies (10.8%) with an Apgar score less than 7/10 at five minutes. Of these 198 depressed neonates 90 (45.5%) received ventilation with bag-and-mask. In the sample there were 31 intra-partum related deaths, where 30 had an Apgar-score of 0/10 at both one and five minutes. A minority, 11/31 (35.5%), of the intra-partum related deaths received ventilation with bag-and-mask, whereas suction of the airways was applied in 16/31 (51.6%).When cross-comparing the four separate observational record forms that were completed for each randomized CCD-camera recording, it was found that the inter- and intra-rater reliability varied considerably amongst the different variables analyzed. The variables that had the highest correspondence across all forms where Outcome, Bag & Mask total time, Bag-and-mask time intervals, Bag-and-mask yes/no, and Suction yes/no, all matching in over 91% (91.3-97.8%) of the forms (Figure 2).

## Discussion

Similar to other published work on the use of CCD-cameras as a method to evaluate neonatal resuscitation, this observational study found this technique to be useful for identifying deviations from protocol and areas for improvement [6, 8–10]. It is reasonable to assume that it could also be applicable to other evaluation situations, not only in low-income countries, but in a variety of settings.

Assessing the resuscitation routines at Paropakar Maternity and Women’s Hospital found that suction was used in almost 90% of newborns brought to the resuscitation table, whereas bag-and-mask ventilation was only used in less than 10%. According to both the national guidelines and the hospital’s own protocol, suction and bag-and-mask ventilation should be performed in all cases where the baby is not breathing at birth, which gives a high adherence to the guidelines in terms of use of suction, but a very low when considering the use of bag-and-mask ventilation. The guidelines for neonatal resuscitation at PMWH are very similar to the WHO’s international guidelines. Both state that after no more than 30 seconds of initial drying, stimulation, clearing of airways and evaluation, bag-and-mask ventilation should be performed [5].

WHO recommendations on basic newborn resuscitation states that: ‘In neonates born through clear amniotic fluid who start breathing on their own after birth, suctioning of the mouth and nose should not be performed’. Strength of this recommendation is strong and the quality of evidence is high. They also state that suctioning of the mouth and nose of newborns that are not breathing at birth and are born through clear amniotic fluid should not be done routinely before initiating bag-and-mask ventilation, although the strength of this recommendation is weak due to lack of published evidence [4]. Several studies recently conducted have shown that routine suctioning is associated with lower five-minute Apgar-scores, lower oxygen saturation levels, and bradycardia [4, 15]. The fact that suction was used for a large number of the cases where the baby was breathing satisfactory when placed on the resuscitation table shows that there is a lot of room for improvement in terms of the use of suction at the hospital.

### Limitations

There were several limitations to the technique used in this study that made assessing the variables included in the observational forms difficult, and therefore affecting the reliability of the observational forms in a negative way. We have chosen only to present the variables showing a good inter-rater reliability. When analysing the recorded resuscitations the baby’s condition was often hard to assess, particularly in the films where the picture quality was far from optimal. The most sensitive indicator of resuscitation being successful is an increase in the baby’s heart rate, which could be assessed using pulse oximeters. Assessment of pulse should be done regularly for babies requiring resuscitation [15], and should according to both international guidelines and the hospital’s protocol be evaluated within the first 30 seconds. In the cases studied, the baby’s heart rate was rarely checked at any point during the resuscitation. In this study, pulse oximeters where not used due to technical difficulties in setting them up and coordinating them with the camera recordings. Therefore, neither the staff nor the observers had sufficient information about the baby’s heart rate or oxygenation, something that could have been very valuable when assessing the resuscitation efforts and outcome.

As in many other low-resource hospitals, a large portion of the health personnel working in the birth facility where not qualified skilled birth attendants. Many of the labours stations were run by nursing students, and there were relatively few doctors and supervisors present. This may have contributed to the fact that the neonatal resuscitation routines at the hospital to a great extent were not in accordance with the guidelines.

The scope of this study did not allow for the evaluation of teamwork and communication. This would require separate methods of investigation as well as an alternative application of the CCD-camera technique. However, it is an interesting area of potential research using the CCD camera technique, which could be investigated at a later date.

## Conclusion

CCD camera technique was found to be a feasible method to assess resuscitation practices in this low resource hospital setting. The neonatal resuscitation protocol in place at the hospital did not differ much from the international guidelines for limited-resource settings. In most aspects, the staff did not adhere to these guidelines. The use of bag-and-mask ventilation was inadequate, and both suction and oxygen were given excessively in terms of protocol. Further studies exploring the underlying causes behind the lack of adherence to the neonatal resuscitation guidelines should be conducted in order to improve compliance and increase the possibility of fulfilling Millennium Development Goal (MDG) 4 by 2015.

## Electronic supplementary material

Additional file 1: **CCD observation form. PDF template of the observational form used when registering information from the resuscitation cases recorded by the** **CCD-cameras.** (PDF 381 KB)

## Abbreviations

CCD: Change-coupled device
WHO: World health organization
HBB: Helping babies breathe
PMWH: Paropakar maternity and woman’s hospital
PMR: Perinatal mortality rate
MNSC: Maternal and newborn service centre
CSPro: Census and survey processing system
SPSS: Statistical package for the social sciences
MDG: Millennium development goal.
