# Supplementary material for: Poor adherence to neonatal resuscitation guidelines exposed; an observational study using camera surveillance at a tertiary hospital in Nepal
Source: BMC Pediatr. 2014 Sep 16;14:233. doi: 10.1186/1471-2431-14-233 (PMC4176581; doi:10.1186/1471-2431-14-233)
Supplement: Supplementary file 1 — Additional file 1: CCD observation form. PDF template of the observational form used when registering information from the resuscitation cases recorded by the CCD-cameras. (PDF 381 KB) [file 12887_2014_1156_MOESM1_ESM.pdf]

Perinatal Mortality Study				FORM-D
**CCD OBSERVATION FORM (FORM-D)**				
**Data ID**	**Observation**	**Write and circle when applicable**	**Write and circle when applicable**	**Write and circle when applicable**
	**This information from 101 to 113 is entered after observing recorded CCD**			
	**ID no.**			
	**Admission number**			
**101**	**Place of Table**	Emergency................ 1<br>Operation theatre... 2<br>Labour Room............3<br>MNSC........................ 4<br>Other..........................	Emergency................1<br>Operation theatre... 2<br>Labour Room............3<br>MNSC........................ 4<br>Other..........................	Emergency................ 1<br>Operation theatre... 2<br>Labour Room............3<br>MNSC........................ 4<br>Other..........................
**102**	**Date of resuscitation (dd/mm/yyyy)**	____/____/_______	____/____/_______	____/____/_______
**103**	**Time of baby bringing to table**	_____/_____	_____/_____	_____/_____
**104**	**Baby crying**	Yes............ 1<br>No............. 2	Yes............ 1<br>No............. 2	Yes............ 1<br>No............. 2
**105**	**Baby resuscitated with stimulation**	Yes............ 1<br>No............. 2	Yes............ 1<br>No............. 2	Yes............ 1<br>No............. 2
**106**	**Duration**	_______ to_______	_______ to_______	_______ to_______
**107**	**Baby resuscitated with Suction**	Yes............ 1<br>No............. 2	Yes............ 1<br>No............. 2	Yes............ 1<br>No............. 2
**108**	**Duration**	_______ to_______	_______ to_______	_______ to_______
**109**	**Baby provided oxygen**	Yes............ 1<br>No............. 2	Yes............ 1<br>No............. 2	Yes............ 1<br>No............. 2
**110**	**Duration**	_______ to_______	_______ to_______	_______ to_______
**111**	**Baby resuscitated with Bag and Mask**	Yes............ 1<br>No............. 2	Yes............ 1<br>No............. 2	Yes............ 1<br>No............. 2
**112**	**Duration**	_______ to_______	_______ to_______	_______ to_______
**113**	**Time of first cry**	_____/_____	_____/_____	_____/_____
**114**	**Outcome of baby**	Live................. 1<br>F. Still birth ......2<br>M. Still birth......3<br>Dead.................4	Live................. 1<br>F. Still birth ......2<br>M. Still birth......3<br>Dead.................4	Live................. 1<br>F. Still birth ......2<br>M. Still birth......3<br>Dead.................4
**115**	**Referral of the baby**	Yes............ 1<br>No............. 2	Yes............ 1<br>No............. 2	Yes............ 1<br>No............. 2
**Matching with CCD record with Case record form B and transfer information from Case record form B of the baby**				
**116**	**Mothers Name**			
**117**	**Time of Birth**	_____/_____	_____/_____	_____/_____
**118**	**Place of Birth**	Emergency................ 1<br>Operation theatre... 2<br>Labour Room............3<br>MNSC........................ 4<br>Other..........................	Emergency................1<br>Operation theatre... 2<br>Labour Room............3<br>MNSC........................ 4<br>Other..........................	Emergency................ 1<br>Operation theatre... 2<br>Labour Room............3<br>MNSC........................ 4<br>Other..........................
**119**	**Date of Delivery**	____/____/_______	____/____/_______	____/____/_______
**120**	**Gestational Age of baby**	_____________ weeks	_____________ weeks	_____________ weeks
**121**	**APGAR at 1 min**			
**122**	**APGAR at 5 min**			
**123**	**Birth weight**	_____________ grams	_____________ grams	_____________ grams
**124**	**Sex**	Male............ 1<br>Female......... 2	Male............ 1<br>Female......... 2	Male............ 1<br>Female......... 2
**125**	**Name of surveillance officer**			
